# MAPK p38alpha Kinase Influences Haematopoiesis in Embryonic Stem Cells

**DOI:** 10.1155/2019/5128135

**Published:** 2019-06-02

**Authors:** Kateřina Štefková, Markéta Hanáčková, Jan Kučera, Katarzyna Anna Radaszkiewicz, Barbora Ambrůžová, Lukáš Kubala, Jiří Pacherník

**Affiliations:** ^1^Institute of Experimental Biology, Faculty of Science, Masaryk University, Kotlářská 2, 611 37 Brno, Czech Republic; ^2^Institute of Biophysics, Academy of Sciences of the Czech Republic, Královopolská 2590/135, 61200 Brno, Czech Republic

## Abstract

The activation of p38alpha kinase mediates cell response to various extracellular factors including many interleukins and growth factors important for haematopoiesis. The role of p38alpha kinase was previously analysed in particular haematopoietic cells. In this study and for the first time, the role of p38alpha kinase in haematopoiesis was studied using a model of continuous haematopoietic development in pluripotent embryonic stem cells *in vitro*. The expression of transcripts associated with haematopoiesis and the potential for the formation of specific haematopoietic cell colonies were compared between wild-type and mutant p38alpha gene-depleted cells. The absence of p38alpha kinase led to the inhibition of hemangioblast formation during the first step of haematopoiesis. Later, during differentiation, due to the lack of p38alpha kinase, erythrocyte maturation was impaired. Mutant p38*α*−/− cells also exhibited decreased potential with respect to the expansion of granulocyte colony-forming units. This effect was reversed in the absence of erythropoietin as shown by colony-forming unit assay in media for colony-forming unit granulocytes/macrophages. p38alpha kinase thus plays an important role in the differentiation of common myeloid precursor cells into granulocyte lineages.

## 1. Introduction

Embryonic stem (ES) cells are derived from pluripotent cells of the inner cell mass of a blastocyst and have the potential to turn into cells of all three germ layers in the body. The differentiation of ES cells therefore represents a unique *in vitro* model for the analysis of developmental processes. The hematopoietic specification of ES cells has been shown to recapitulate embryonic haematopoiesis [1, 2]. Haematopoiesis in embryonal development represents a complex of developmental process that involves several anatomical sites, after which HSCs that have finally arisen colonise bone marrow at birth. The first wave of haematopoiesis takes place in the yolk sac, the extraembryonic organ of the embryo, and is called primitive haematopoiesis. Nucleated so-called primitive erythrocytes, which have the embryonal type of hemoglobin, appear in the yolk sac along with some myeloid precursors. The second wave, already referred to as definitive, is rapidly followed by the emergence of erythromyeloid progenitors (EMP) and lymphocytes also in the yolk sac. The third wave occurs in the intraembryonic AGM (aorta-gonad-mesonephros) area, and definitive HSCs originate here from hemogenic endothelium [3–5]. Then, the HSCs migrate into the fetal liver, which serves as the main hematopoietic organ of the fetus [6].

Mitogen-activated protein kinases (MAPK) are a superfamily of protein kinases that are the key players in numerous signaling events in cells from yeast to mammals. The MAPK superfamily comprises at least four families, namely, extracellular signal-related kinases 1 and 2 (ERK1 and ERK2), ERK5, Jun amino-terminal kinases (JNKs), and p38 kinases. Kinase p38 has been characterised as a protein kinase which is activated in mammalian cells in response to lipopolysaccharide, toxins, radicals, and extracellular changes in osmolarity, linking the p38 kinase pathway to a stress-induced response. Moreover, it has been shown that p38 kinase is involved in many other cellular responses including cell proliferation, differentiation, development, and apoptosis.

Four isoforms of p38 kinase have been identified so far: p38*α*, p38*β*, p38*δ*, and p38*γ* [7, 8]. In the majority of cell types, p38*α* is the most abundant p38 family member. Kinase p38*α* has a key role in the regulation of developmental processes as has been demonstrated in animal models. It is known that in adults, p38*α* kinase is required for HSC activation as well as for the specification and maturation of hematopoietic cell lineages [9]. During mouse embryogenesis, the depletion of p38*α* kinase leads to embryonic mortality at around E10.5 due to defects in vascularisation and in the formation of vessel structures in placenta [10–13]. In one study, it was also found that if p38*α*−/− embryos survived up to 16.5 dpc, they were also anemic due to abnormal erythropoiesis, which is caused by the insufficient production of erythropoietin [14]; however, in this case, other hematopoietic cell lineage developments were not investigated. In addition, studies performed with p38 knockout ES cells or with the biochemical inhibition of p38 kinase showed that p38 controls mesodermal commitment during ES cell differentiation [15]. Therefore, we hypothesized that p38*α* kinase plays a role in the development of hemangioblast and its differentiation into hematopoietic lineages.

Our results show that p38*α* affects haematopoiesis in at least three different ways. Firstly, p38*α* is required for hemangioblast formation *in vitro*. Secondly, p38*α* is required for erythropoiesis and erythrocyte maturation. Finally, p38*α* regulates the differentiation of common myeloid progenitor (CMP) cells into granulocyte lineages.

## 2. Materials and Methods

### 2.1. Culture and Differentiation of ES Cells

In this study, cells deficient in p38*α* kinase (p38*α*−/−) and their wild-type counterpart (p38*α*+/+) were used (kindly provided by Dr. Barry P. Sleckman, Washington University School of Medicine at St. Louis). The generation of these cell lines is described in detail by Kim and coworkers [16]. The ES cells were maintained in an undifferentiated state in a monolayer on a gelatinized dish (by 0.1% water solution of porcine gelatin) in Dulbecco's modified Eagle's medium supplemented with 15% fetal calf serum, 100 mM nonessential amino acids (all Gibco-Invitrogen, UK), 0.05 mM *β*-mercaptoethanol (Sigma-Aldrich, USA), 100 U/ml penicillin, 0.1 mg/ml streptomycin (Gibco-Invitrogen, UK), and 1000 U/ml recombinant leukemia inhibitory factor (LIF) (Chemicon International, USA). The differentiation of the cells was induced spontaneously through the formation of embryoid bodies (EBs), floating cell aggregates, and LIF depletion. The formation of embryoid bodies was achieved by the hanging drop technique (400 cells/drop) or by the direct culture of ES cells on bacteriological dishes coated with agar (0.5% agar diluted in water, 5 ml per 90 mm in diameter dish) in complete ES medium without LIF (5 × 10^6^ cells per 90 mm in diameter dish). The medium was replaced every two days.

### 2.2. Quantitative RT-PCR Analysis of Gene Expression

In order to identify hematopoietic processes, the expressions of selected transcription factors associated with haematopoiesis were analysed. The expressions of the following genes were used: (i) VEGF, Flk-1, Etv2, GATA2, Tal1, Runx1, Sca1, c-Kit, and Tie2 as markers of hemangioblast/early haematopoiesis, (ii) HoxB4, CD34, CD38, and CD150 as markers of haematopoiesis, (iii) GATA1, Klf1, EpoR, Hbb *ζ*, Hbb-b1, and Hbb *γ* as markers of erythroid lineage, (iv) PU.1, C/EBP*α*, G-CSF-R (v1, v2), and csfr1m as markers of myeloid lineage, and (v) the key hematopoietic cytokines IL6, IL3, and EPO (Table 1).

Total RNA was extracted by UltraClean® Tissue & Cells RNA Isolation Kit (MO BIO Laboratories, USA). cDNA was prepared using Mu-MLV reverse transcriptase kit (Sigma-Aldrich, USA). qRT-PCR was performed in a Roche LightCycler using the following program: an initial activation step at 95°C for 5 min, followed by 40 cycles at 95°C for 10 s, an annealing temperature (see Table 1) for 10 s, and a temperature of 72°C for 10 s.

### 2.3. Immunoblot Analysis

Immunoblot analysis and cell sample harvesting were performed as presented previously [40]. Briefly, ES cells and/or EBs were washed twice with PBS (pH 7.2) and lysed in sodium dodecyl sulphate (SDS) lysis buffer (50 mM Tris-HCl, pH 7.5; 1% SDS; 10% glycerol). Protein concentrations were determined using the DC protein assay kit (Bio-Rad, USA). Lysates were supplemented with bromophenol blue (0.01%) and *β*-mercaptoethanol (1%) and incubated for 5 min at 95°C. Equal amounts of total protein (10 *μ*g) were subjected to 8 or 10% SDS-PAGE. After being electrotransferred onto polyvinylidene difluoride membrane (Immobilon-P, Millipore, USA), proteins were immunodetected using appropriate primary and secondary antibodies and visualized by ECL Plus reagent (Amersham Pharmacia Biotech, USA) according to manufacturer's instructions. We used the following primary antibodies: rabbit polyclonal antibodies against p38alpha kinase, Oct4 (Santa Cruz Biotechnology, USA), phospho-p38 kinase, and GAPDH (Cell Signaling Technology, USA). After immunodetection, each membrane was stained by Amido black to confirm equal protein loading. The total level of *β*-actin was detected as loading control.

### 2.4. Colony-Forming Assay

The formation of ES cells into EBs was achieved by the direct culture of ES cells on bacteriological dishes, as described above. After 3 (for hemangioblast colonies), 6, 10, or 14 (for CFU colonies) days of differentiation, EBs were dissociated into single cells and 3 × 10^4^ cells were replated into 1.0% methylcellulose-based medium containing a cocktail of hematopoietic cytokines (MethoCult; STEMCELL Tech., Canada). After 3 (for hemangioblast colonies) or 14 (for CFU colonies) days of cultivation, hematopoietic colonies were scored and their morphologies were documented by photography [41].

To analyse the efficiency of the differentiation into hematopoietic progenitors, we used colony-forming assay in various MethoCult media. The cells were seeded into methylcellulose with cytokines specific to erythroid and myeloid differentiation, indicated as full medium (MethoCult GF M3434, containing SCF, IL-3, IL-6, and EPO), erythroid differentiation (MethoCult GF M3334, containing EPO, indicated as erythroid media), or granulocyte and macrophage differentiation (MethoCult GF M3534, containing SCF, IL-3, and IL-6, indicated as GM media). For blast colonies, cells were seeded into full media supplemented with 5 ng per ml of mouse VEGF (PeproTech, USA). The numbers and morphologies of formed colonies were examined by means of light microscopy.

### 2.5. Identification of Hemoglobin-Positive Cells

The formation of ES cells into EBs was achieved by the hanging drop technique. After 5 days of differentiation, EBs were transferred into 24-well plates previously coated with 0.1% gelatin (1 EB per 1 well). As described above, the EBs were then cultured in DMEM-F 12 (1 : 1) supplemented with insulin, transferrin, selenium (ITS; Gibco-Invitrogen, UK), and antibiotics, which was then used as serum-free medium. The medium was replaced every two days. EBs with red cell islands were scored under a microscope. The 6-, 10-, and 14-day-old adhesive EBs were stained with 2,7-diaminofluorene (DAF) (Sigma-Aldrich, USA) (0.1% DAF, 0.1% H_2_O_2_, and 200 mM Tris-HCl pH 7). Due to the pseudoperoxidase activity of hemoglobin, erythroid cells oxidise DAF, which catalyzes the formation of a blue compound (fluorene blue). Blue cells were then observed under the microscope.

### 2.6. Quantitative Staining of Hemoglobin by DAF

EBs were prepared in suspension as described earlier. After 6, 10, or 14 days of differentiation, the EBs were washed twice with cold PBS. Then, the EBs were lysed in NP-40 lysis buffer and frozen at -20°C. 50 *μ*l of lysates and 150 *μ*l of assay buffer (0.1% DAF, 0.06% H_2_O_2_, 100 mM Tris phosphoric acid buffer pH 7, and 6 M Urea) were pipetted into a 96-well plate and absorbance was measured at 620 nm.

### 2.7. Statistics

Data are expressed as mean ± SD. Statistical analysis was performed using ANOVA or the Kruskal-Wallis test with post hoc Bonferroni or Dunn's test. Values of *P* < 0.05 were considered to be statistically significant.

## 3. Results

### 3.1. p38*α* MAPK Regulates Haematopoiesis in ES Cells

The general/overall hematopoietic potential of wt and mutant p38*α*−/− ES cells was analysed both by an assay of the potential to form hematopoietic colony-forming unit (CFU) cells and by determination of the expression of transcripts which are associated with haematopoiesis (see Materials and Methods).

**Figure 1:**
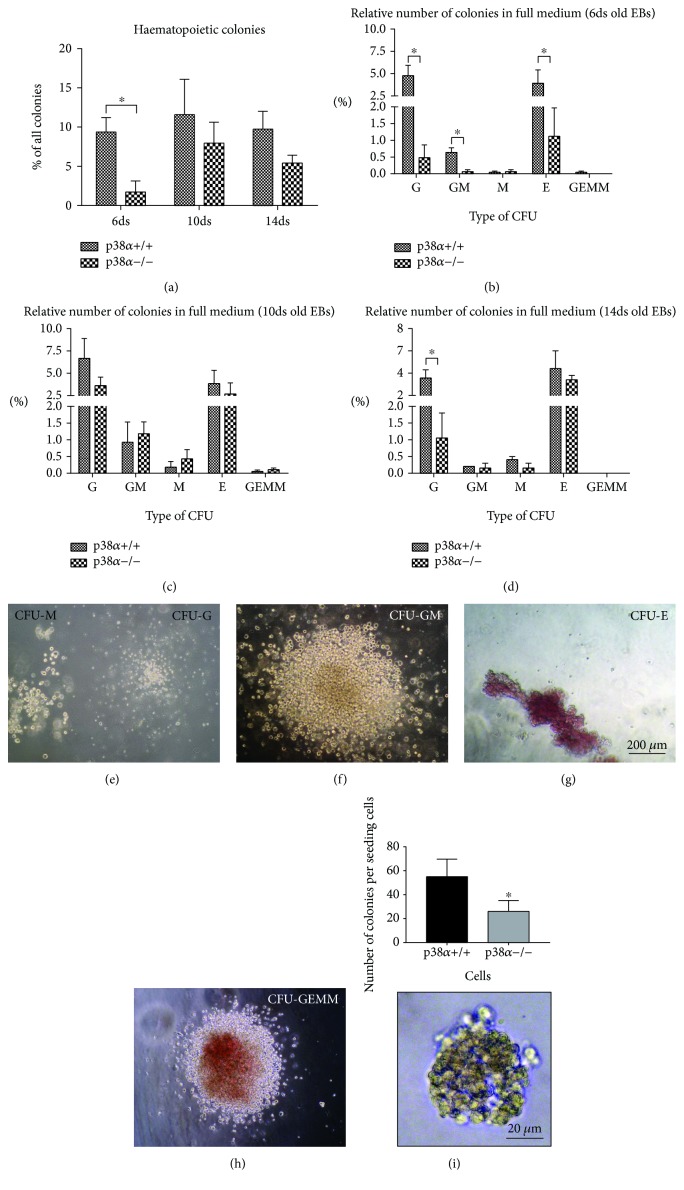
Formation of hematopoietic CFU in wild-type and mutant p38*α*−/− EBs on days 6, 10, and 14 of differentiation. Single-cell suspensions were seeded into complete hematopoietic selective media for 14 days. The overall frequency of all types of hematopoietic colonies (a) and the frequencies of particular CFU-G, CFU-GM, CFU-M, CFU-E, and CFU-GEMM on days 6 (b), 10 (c), and 14 (d) are shown. Representative morphologies of the determined hematopoietic CFU colonies, CFU-M and CFU-G (e), CFU-GM (f), CFU-E (g), and CFU-GEMM (h) are presented. The formation of hemangioblast progenitor/blast cell colonies and their representative morphology are also shown (i). Data are presented as mean + SEM from a minimum of four independent experiments. Statistical significance was determined by ANOVA with post hoc Bonferroni's multiple comparison test; asterisk “^∗^” indicates a statistical significance of *P* < 0.05.

The full medium for the CFU, which supports all expected types of hematopoietic CFU, was used in this study. When 6-day-old EBs were used, p38*α*−/− cells formed a lower number of hematopoietic CFU than their wt counterparts. In 10- and 14-day-old EBs, we did not observe any difference in the formation of hematopoietic CFU (Figure 1(a)). In detail, five types of CFU were recognised [42]: CFU-M (macrophage) and CFU-G (granulocyte; Figure 1(e)), CFU-GM (granulocyte, macrophage; Figure 1(f)), CFU-E/BFU-E (erythrocyte/burst-forming unit erythroid; Figure 1(g)), and CFU-GEMM (granulocyte, erythrocyte, macrophage, and megakaryocyte; Figure 1(h)). CFU-G and CFU-E/BFU-E were formed at a higher frequency in about 5% of colonies. CFU-GM was formed in about 1% of colonies derived from 6- and 10-day-old EBs. The count of CFU-M and CFU-GEMM was under 0.5% in all cases. An overall higher hematopoietic CFU capacity/potential was observed in 10-day-old EBs (Figure 1(a)). Not only does the absence of p38*α* in cells lead to a decrease in overall hematopoietic CFU capacity (the count of all CFU mentioned above) but also 6-day-old p38*α*−/− EBs have a lower number of CFU-G, CFU-GM, and CFU-E in contrast to their wt counterparts (Figure 1(b)). No difference was observed between p38*α*+/+ and p38*α*−/− in the number of particular hematopoietic CFU in 10- and 14-day-old EBs; however, there was a decrease in CFU-G in 14-day-old p38*α*−/− EBs (Figures 1(c) and 1(d)). Hemangioblast progenitor/blast cell colonies were also determined. Mutant p38*α*−/− cells formed a lower number of blast colonies than their wt counterparts (Figure 1(i)).

**Figure 2:**
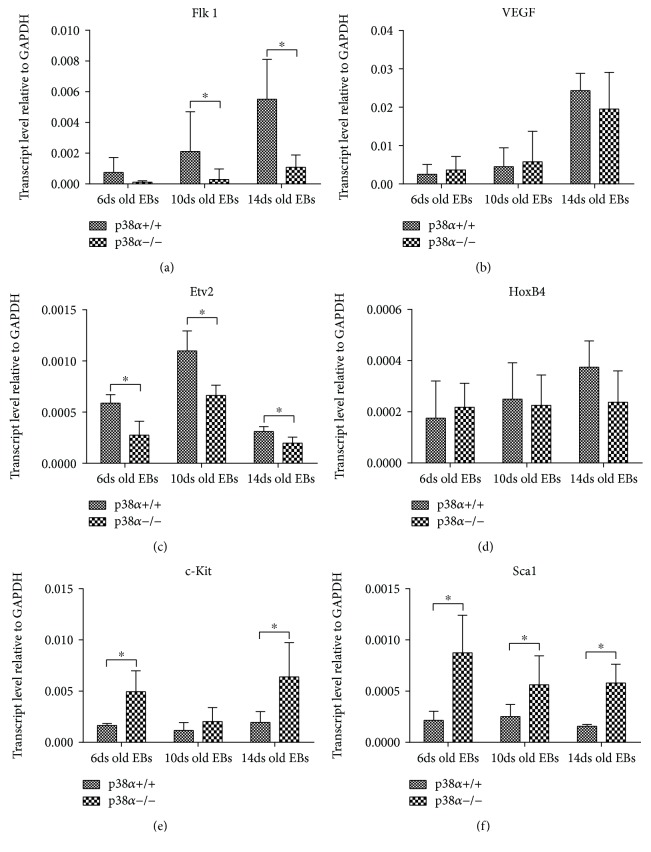
The expressions of genes required for and/or marking the development of hemangioblast and early hematopoietic development determined by qRT-PCR. The levels of transcripts of key components of the Flk1 signaling axis, Flk1 (a), VEGF (b), and Etv2 (c) and the levels of transcripts of hemangioblast/hematopoietic markers of HoxB4 (d), cKit (e), and Sca1 (f) in 6-, 10-, and 14-day-old EBs, are shown. Data are presented as mean + SEM from a minimum of four independent experiments. Statistical significance was determined by ANOVA with post hoc Bonferroni's multiple comparison test; asterisk “^∗^” indicates a statistical significance of *P* < 0.05.

**Figure 3:**
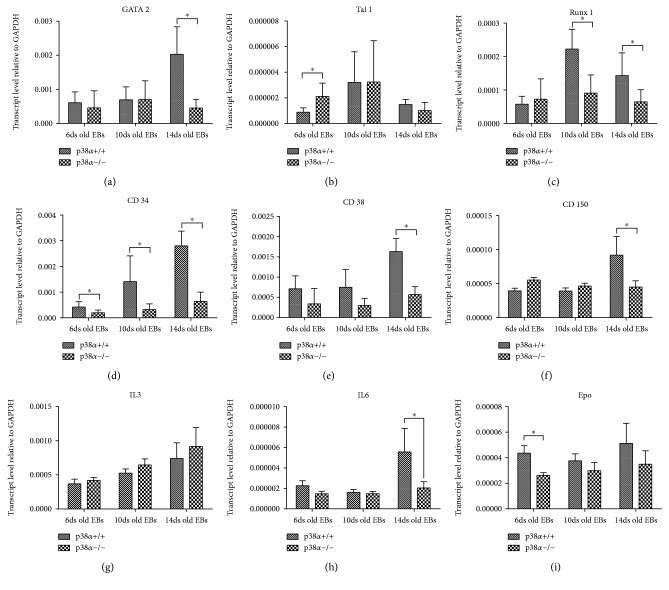
The expressions of some transcripts of haematopoiesis-regulating transcription factors, hematopoietic cell markers, and key hematopoietic cytokines determined by qRT-PCR. The levels of transcription factors GATA2 (a), Tal1 (b), and Runx1 (c), the transcripts of hematopoietic markers CD34 (d), CD38 (e), and CD150 (f), and the transcripts of hematopoietic key cytokines IL3 (g), IL6 (h), and EPO (i) in 6-, 10-, and 14-day-old EBs are shown. Data are presented as mean + SEM from a minimum of four independent experiments. Statistical significance was determined by ANOVA with post hoc Bonferroni's multiple comparison test; asterisk “^∗^” indicates a statistical significance of *P* < 0.05.

Further, we determined the expression of various transcripts associated with hematopoietic differentiation in samples of 6-, 10-, and 14-day-old EBs by means of qRT-PCR. A wide range of genes related to this process, from genes linked to mesoderm/hemangioblast to genes which play a role in primitive and definitive haematopoiesis, was chosen for analysis. The expression of Flk1 increased during differentiation and was reduced in mutant p38*α*−/− cells (Figure 2(a)). Flk1 ligand VEGF expression was also increased during differentiation, but no difference between wt and mutant cells was observed (Figure 2(b)). In contrast to Flk1 and VEGF, Etv2 is expressed transiently in a narrow developmental window (E7–E9.5 *in vivo*) [23]. Our results showed that the expression of Etv2 was higher in 10-day-old EBs, compared to 6- and 14-day-old EBs (Figure 2(c)). Compared to mutant p38*α*−/− cells, wt cells exhibited a higher level of Etv2 transcripts at all determined times of differentiation. Next, we analysed the expression of key regulators of hematopoietic development. The level of HoxB4 transcription was similar for each time and for both genotypes (Figure 2(d)). Interestingly, the expressions of c-Kit and Sca1 transcripts were higher in mutant p38*α*−/− cells, except for the level of c-Kit in 10-day-old EBs, where it was lower and comparable in both cell types (Figures 2(e) and 2(f)). The expression of GATA2 was higher in 14-day-old wt EBs. In mutant p38*α*−/− cells as well as in 6- and 10-day-old wt EBs, the GATA2 level was low and comparable to that in wt cells in 6- and 10-day-old EBs at all times of analysis (Figure 3(a)). Tal1 expression was higher in mutant p38*α*−/− than in wt 6-day-old EBs. In older EBs, its level in wt was identical to that in mutant EBs (Figure 3(b)). The level of Runx1 was increased in 10- and 14-day-old wt EBs compared to both 6-day-old wt EBs and mutant EBs at all times of analysis (Figure 3(c)). The expression of CD34 increased continuously during differentiation in wt EBs but was lower in mutant EBs than in wt, and its level was not elevated during mutant EB differentiation (Figure 3(d)). In contrast to CD34, the expressions of CD38 and CD150 were significantly upregulated in 14-day-old wt EBs. In 6- and 10-day-old EBs, the expressions of CD38 and CD150 were low, independent of cell genotype, and also corresponded to the levels of particular transcripts in 14-day-old p38*α*−/− EBs (Figures 3(e) and 3(f)). The expressions of IL3, IL6, and EPO transcripts, key hematopoietic cytokines [21, 33], were also determined. With the exception of IL6 on day 14 and EPO on day 6 of differentiation, we did not observe differences in the levels of these transcripts between wt and p38*α*−/− cells. The expression levels of IL6 and EPO transcripts at these times were lower in p38*α*−/− cells compared to their wt counterparts (Figures 3(g)–3(i)).

Thus, p38*α* kinase is required for regular haematopoiesis. Pluripotent p38*α*−/− ES cells have an attenuated potential for the formation of hematopoietic CFU. This corresponds with the low expressions of transcripts Flk1, Etv2, and Runx1, which are key regulators of haematopoiesis.

### 3.2. p38*α* Kinase Is Required for Erythropoiesis

**Figure 4:**
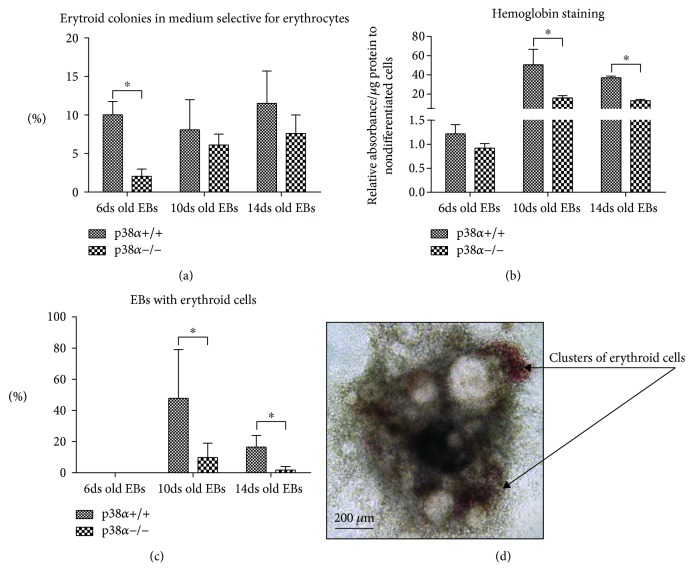
Analyses of erythropoiesis. Single-cell suspensions from 6-, 10-, and 14-day-old EBs were seeded into erythroid CFU selective media for 14 days. The frequency of CFU-E/BFU-E in wild-type and mutant p38*α*−/− EBs is shown (a). We also determined the hemoglobin level based on their pseudoperoxidase activity (b) and the frequency of EBs with visible erythroid clusters in wild-type and mutant p38alpha cells (c). A representative EB with red-coloured clusters of erythroid cells is also shown (d). Data are presented as mean + SEM from a minimum of four independent experiments. Statistical significance was determined by ANOVA with post hoc Bonferroni's multiple comparison test; asterisk “^∗^” indicates a statistical significance of *P* < 0.05.

Erythropoiesis itself was also studied in detail. The overall number of erythroid colonies in erythroid-specific CFU media was not different in wt cells derived from 6-, 10-, or 14-day-old EBs. Mutant p38*α*−/− cells formed a lower number of CFU-E in comparison with their wt counterparts when cells were seeded from 6-day-old EBs. We observed no difference in the number of CFU-E/BFU-E between wt and p38*α*−/− cells derived from 10- and 14-day-old EBs (Figure 4(a)). The reduced erythropoietic potential of p38*α*−/− cells was further confirmed by the low hemoglobin level determined by staining for pseudoperoxidase activity. The hemoglobin level was near to the assay background in 6-day-old EBs but strongly increased in 10- and 14-day-old EBs (Figure 4(b)), which correlates with the observable clusters of erythroid cells in differentiating EBs. The first rare red-coloured erythroid cell clusters were observed from day 8 or day 9 of differentiation (not shown). In wt p38*α*+/+ cells, about 50% of EBs were contained from 1 to 3 red-coloured clusters of erythroid cells. They were clearly visible up to days 12 or 13 of differentiation. Later, these clusters disappeared. They were present in only 20% of 14-day-old differentiating EBs. In mutant p38*α*−/− cells, no more than 10% and 2% of EBs were positive for these erythroid clusters on days 10 and 14 of differentiation, respectively (Figures 4(c) and 4(d)).

**Figure 5:**
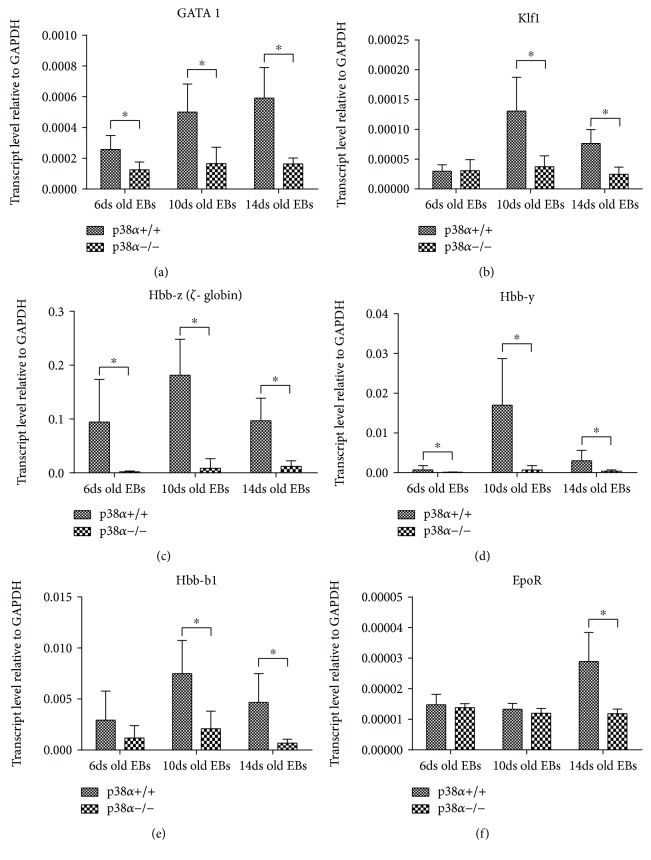
Expression of erythropoiesis-regulating transcription factors and markers determined by qRT-PCR. The levels of transcription factors GATA1 (a) and Kfl1 (b) and the transcripts of hemoglobin Hbb *ζ* (c), Hbb *γ* (d), Hbb-b1 (e), and EpoR (f) are shown in wild-type and mutant p38*α*−/− cells. Data are presented as mean + SEM from a minimum of four independent experiments. Statistical significance was determined by ANOVA with post hoc Bonferroni's multiple comparison test; asterisk “^∗^” indicates a statistical significance of *P* < 0.05.

We also determined the expressions of transcripts which play a key role in erythropoiesis (GATA1 and Klf1) and of globin transcripts (Hbb-b1, *ζ*-globin, and *γ*-globin). wt p38*α*+/+ cells exhibited higher levels of GATA1 and Klf1 transcripts than mutant p38*α*−/− cells (Figures 5(a) and 5(b)). The analysis of hemoglobin transcript expressions showed similar results: lower levels of Hbb-b1, *γ*- (gamma-) globin, and *ζ*- (zeta-) globin transcripts were observed in p38*α*−/− cells compared to p38*α*+/+ cells (Figures 5(c)–5(e)). Interestingly, there was no difference in the levels of EpoR transcripts between wt and mutant cells, except on day 14, when its expression was higher in wt cells (Figure 5(f)).

The erythroid lineage, CFU-E, was significantly reduced in p38*α*−/− cells at the early phase of haematopoiesis, which corresponds to the reduction in hemangioblast progenitors (see above). Later, the number of CFU-E was the same in both cell lineages but p38*α*−/− erythrocytes did not mature, as is shown by the reduced levels of GATA1, hemoglobin transcripts, and hemoglobin protein.

### 3.3. Involvement of p38*α* in Myeloid Differentiation

Next, the differentiation into myeloid lineages was studied in detail. First, cells from 6-, 10-, and 14-day-old EBs were tested for granulocyte and monocyte colony-forming potential in granulocyte-monocyte- (GM-) selective media. CFU-G represented about 8% of all colonies formed from wt 6- and 10-day-old EBs. Its proportion decreased to 4% in wt 14-day-old EBs.

**Figure 6:**
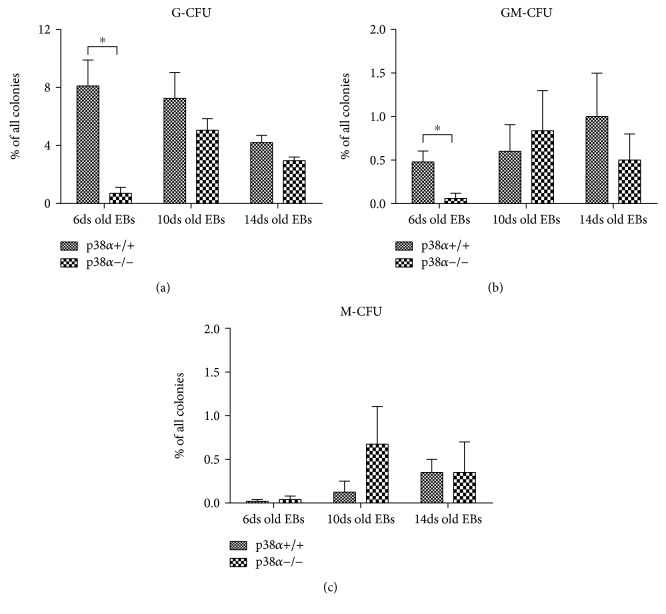
Formation of CFU-G, CFU-GM, and CFU-M colonies in myeloid CFU selective media. Wild-type (p38*α*+/+) and mutant (p38*α*−/−) 6-, 10-, and 14-day-old EBs were dissociated into single cells by trypsin and seeded for 14 days into a medium selective for myeloid cells. The frequencies of CFU-G (a), CFU-GM (b), and CFU-M (c) colonies derived from wild-type and mutant p38*α*−/− EBs are shown. Data are presented as mean + SEM from a minimum of four independent experiments. Statistical significance was determined by ANOVA with post hoc Bonferroni's multiple comparison test; asterisk “^∗^” indicates a statistical significance of *P* < 0.05.

Except for p38*α*−/− cells from 6-day-old EBs, the number of CFU-G in mutant p38*α*−/− EBs was similar to that in their wt counterparts. p38*α*−/− cells isolated from 6-day-old EBs exhibited 20 times lower potential to form granulocyte colonies than wt cells (Figure 6(a)).

Colonies of CFU-GM and CFU-M were formed with frequencies of about 1% and 0.5%, respectively. Mutant p38*α*−/− cells formed a lower number of CFU-GM in 6-day-old EBs, but there was no difference in 10- and 14-day-old EBs compared to wt cells. Generally, CFU-M were formed at very low frequencies, which slightly increased up to 0.5% in 10- and 14-day-old EBs (Figures 6(b) and 6(c)). The role of p38*α* in this process remains to be clarified.

**Figure 7:**
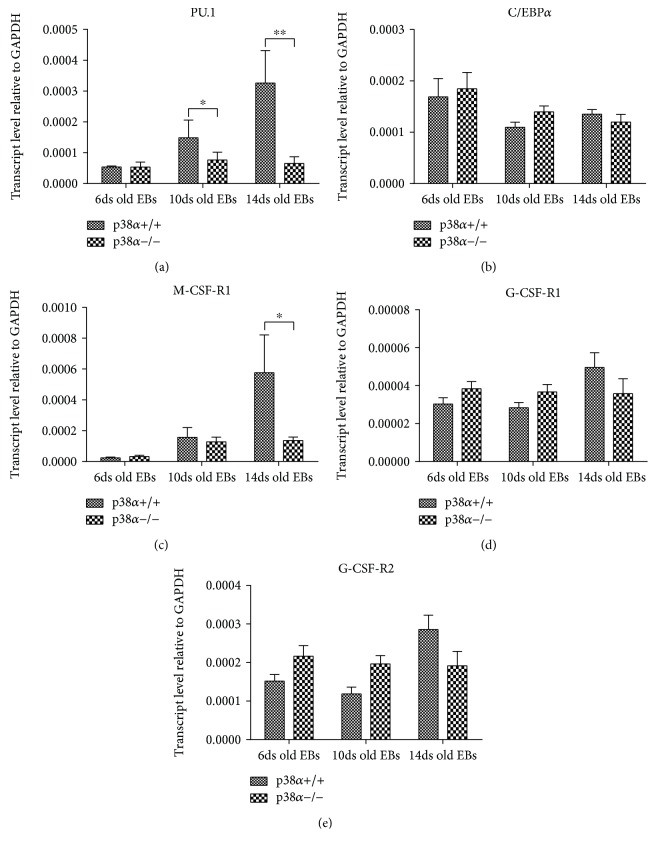
Expression of transcription factors and marker transcripts associated with the regulation of myelopoiesis. Levels of selected transcripts were determined by qRT-PCR in 6-, 10-, and 14-day-old wild-type and mutant p38*α*−/− EBs. The effect of p38*α* depletion on the expressions of transcription factors PU.1 (a) and C/EBP*α* (b) and the receptors coding the transcripts of key myeloid cytokines M-CSF-R1 (c), G-CSF-R1 (d), and G-CSF-R2 (e) is shown. Data are presented as mean + SEM from a minimum of four independent experiments. Statistical significance was determined by ANOVA with post hoc Bonferroni's multiple comparison test; asterisk “^∗^” indicates a statistical significance of *P* < 0.05.

In contrast to the results for G/GM/M colony-forming assay, the levels of key myeloid transcription factors changed due to p38*α* depletion. The expression of PU.1 transcript increased during wt EB differentiation in a time-dependent manner. The highest level was determined in 14-day-old EBs. This was not observed in mutant p38*α*−/− EBs, where the expression of PU.1 was similar at all times of analysis and lower in 10- and 14-day-old EBs than in wt EBs (Figure 7(a)). In contrast to PU.1, the level of C/EBP*α* transcript was not elevated in EBs in either a time- or genotype-dependent manner (Figure 7(b)). Also, we did not detect any elevation in G-CSF-R1 or G-CSF-R2 transcripts, although M-CSF-R1 had a higher expression in 14-day-old wt EBs compared to 6- and 10-day-old wt EBs (Figures 7(c)–7(e)).

Kinase p38*α* is required for the regular expression of the PU.1 transcript. Excluding the early phase of haematopoiesis, the depletion of p38*α* kinase did not significantly affect the formation of CFU-G, CFU-GM, or CFU-M in GM media, which is EPO free, in contrast to full media. Therefore, p38*α* kinase and EPO are able to regulate the fate of myeloid progenitors.

## 4. Discussion

The first step in haematopoiesis is the formation of primitive hematopoietic cells from hemangioblasts. The formation of hemangioblast is induced by the VEGF/Flk1/Etv2 signaling axis. The expression of Flk1 ligand VEGF transcripts was unresponsive to the absence of p38*α* kinase, but cells without p38*α* had lower expressions of both Flk1 and its downstream signaling target Etv2. The Flk1-mediated signaling pathway is attenuated not only by low Flk1 transcription but also by the fact that the binding of VEGF to Flk1 leads to the activation of p38*α* kinase, which is responsible for the induction of Etv2 expression [23]. Hemangioblast is thus formed in a p38*α* kinase-dependent manner. The loss of hemangioblast leads to the attenuation of primitive haematopoiesis within the early stage of development. In the next step, Etv2 continuously induces the formation of hemangioblast and its subsequent transformation into angioblast and hematoblast [43]. In hematoblast, Etv2 induces the expression of Tal1 (Scl), Runx1, GATA1, and GATA2, key regulators of haematopoiesis [27, 44, 45]. Defects in the transcription of Flk1, GATA2, Etv2, and Tal1 lead to embryonic lethality due to defective haematopoiesis and vasculogenesis [46–48]. The depletion of GATA2 results in embryonic lethality at E11.5, in part due to anemia [49]. Etv2 mutant embryos are nonviable after E9.5, and these embryos lack hematopoietic and vascular lineages [50]. Our results showed that the expression levels of Flk1, GATA2, and Etv2 were influenced by the depletion of p38*α* MAPK in ES cells. This also correlated with the low number of hemangioblast colonies in p38*α*−/− cells. Tal1 plays an important role in primitive and definitive haematopoiesis and is a direct upstream regulator of Runx1 [39, 51]. The transcription factors Etv2 and Tal1 are involved in the early steps of haematopoiesis *in vivo*; Etv2 is important for hemangioblast differentiation [27] while Tal1 is responsible for erythropoiesis in the yolk sac and the differentiation of hemogenic endothelium [52–54]. Runx1−/− mice die in utero at E12.5, and their fetal liver contains only primitive erythroblasts. Moreover, it has been demonstrated that Runx1 is crucial in endothelial-hematopoietic transition [36–38, 55]. Tal1 cooperates with other factors and increases the expression of GATA1, Runx1, and itself. Landry and colleagues observed that Runx1 expression in the yolk sac is directly regulated by Tal1 [51]. Moreover, the disruption of Runx1 in mice leads to the decreased expression of GATA1 and Klf1 [56]. While the expression of Tal1 was not affected in p38*α*−/− cells in our study, the expression level of Runx1 and thus the expression level of GATA1 were p38*α* dependent and correlated well with Etv2 expression; that is, both the expression levels of Runx1 and GATA1 decreased in a p38*α*-dependent manner. This could indicate a so-far unknown Etv2-independent mechanism behind Tal1 regulation and/or that Tal1 induces Runx1 expression in a p38*α*-dependent manner.

The expression of HoxB4 was independent of both p38*α* kinase and the level of Etv2. HoxB4 is also a key regulator of the hematopoietic lineage and is essential for the maintenance of hematopoietic stem cells [31, 32]. Therefore, we analysed the expression of other markers which can be associated with hematopoietic stem/progenitor cells. Unexpectedly, the expressions of Sca1 and c-Kit were increased in contrast to the overall decrease in haematopoiesis in p38*α*−/− cells but this could be explained as a delay in the differentiation process [14, 57]. In contrast, lower expressions of CD34 transcripts in mutant cells mark a decrease in hematopoietic potential. However, we did not observe a significant difference in the overall potential for hematopoietic CFU formation between wt and mutant cells derived from 10- and 14-day-old EBs. Thus, our results show that p38*α* is important for hemangioblast formation but not for the further formation of EMP, HPC/HSC, or the hematopoietic progenitors themselves, excluding the CFU-G lineage. Subsequently, on the basis of our results, it should be hypothesized that when haematopoiesis is established, p38*α* signaling is a necessary factor in both erythropoietic maturation and the direction of CMP fate. On the basis of our results and the previously published data discussed above, we propose that p38*α* kinase plays a key role in haematopoiesis through the regulation of Etv2 expression. VEGF, through Flk1, induces the expression and activity of Etv2 via p38*α* kinase. Etv2 is an inducer and a crucial regulator of hemangioblast formation and regulates the expressions of other transcription factors that are necessary for hemangioblast and early hematopoietic development. Moreover, the Etv2-induced expression of Flk1 represents a source of positive feedback in hematopoietic mesoderm formation [58]. The absence of p38*α* prevents the induction of Etv2 by VEGF/Flk1 and inhibits the initial process of haematopoiesis.

When we looked at erythropoiesis itself, the formation of CFU-E/BFU-E was affected in 6-day-old p38*α*−/− EBs. The overall reduction in CFU in 6-day-old p38*α*−/− EBs could be caused by the general delay in mesodermal/hemangioblast formation, which is in agreement with Barruet et al. [15]. In addition, we hypothesized that the decreased number of erythroid progenitors in 6-day-old EBs is connected to a reduction in the GATA1 expression level. Weiss and his colleagues observed that GATA1-deficient ES cells are unable to give rise to primitive erythrocytes, while in definitive erythropoiesis, GATA1 plays a role in the maturation of proerythroblast [59, 60]. The formation of CFU-E/BFU-E was not affected by the depletion of p38*α* kinase in 10- and 14-day-old EBs, although GATA1 and Klf1 transcript levels remained continuously low. In addition, GATA1 and Klf1 factors mediated the maturation of erythrocytes in coordination with EPO, when EPO induced phosphorylation and the transcription activity of GATA1 [61]. Previously, it was shown that EPO activity is mediated by p38*α* kinase and that p38*α* kinase is required for EPO mRNA stability and hemoglobin synthesis. Moreover, the inhibition of p38*α* leads to the blocking of the EPO-dependent accumulation of mouse globin chains in erythroid precursors [14, 57]. Also, erythrocyte maturation is impaired not only by defects in EPO/EpoR downstream signaling but also by a low level of EpoR transcript, the expression of which is induced by GATA1 [61]. This can explain our observations that p38*α* attenuated haematopoiesis and that p38*α*−/− erythroblasts did not mature, although EPO was present in the culture media (full media and erythroid media for CFU) and the expression of EPO was not different in wt and p38*α*−/− cells in 10- and 14-day-old EBs. Thus, it seems that the maturation of erythroblast is not connected only with the expression and/or level of EPO in a p38*α*-dependent manner. We conclude that p38*α* should be necessary for erythrocyte maturation but not for the formation of CFU-E/BFU-E during definitive waves of haematopoiesis, which is in agreement with the observation by Tamura and colleagues [14]. The GATA1 level is regulated through Etv2 and Runx1, whose expression is also decreased in a p38*α*-dependent manner, as discussed above and shown in our results. Erythropoiesis is thus regulated by Etv2, Runx1, and GATA1 signaling in a p38*α*-dependent manner. The depletion of p38*α* leads to the attenuation of Etv2 expression due to the insufficient expression of GATA transcription factors, followed by a decline in erythroblast production. Further, the maintenance of GATA factors and their phosphorylation, which is partially mediated by both p38*α* kinase and the presence of EPO, induces the expression of hemoglobin and erythrocyte maturation. Altogether, the depletion of p38 leads to the formation of a low number of immature erythrocytes.

Myelopoiesis is driven by PU.1 and C/EBP*α* transcripts, the expressions of which are induced by Runx1 [36]. Both myeloid lineages, granulocytes, and monocytes, are induced by increasing PU.1 and C/EBP*α* expressions [62]. A balanced ratio of PU.1 and C/EBP*α* leads to granulopoiesis, but a higher expression ratio of PU.1 to C/EBP*α* leads to monocytopoiesis [63]. The ratio of PU.1 to C/EBP*α* increased continuously during the differentiation of wt cells. However, we did not observe the increased formation of CFU-M in wt, nor, conversely, an increase in G-CFU in mutant cells, as could be expected. CFU-G formation was decreased in p38*α*−/− cells expanded in complete CFU full media. However, the expression of M-CSF-R1 was higher in wt compared to mutant cells in 14-day-old EBs, which could indicate a higher population of promonocytic progenitors. There was no difference in G-CSF-R1 or G-CSF-R2 expression or in the expression of their upstream regulator C/EBP*α*. Nevertheless, the frequency of CFU-M was very low in both cell types. Analyses of the potential role of p38*α* kinase in monocytopoiesis would require a different type of experiment.

Interestingly, when we tested the capacity to form CFU in CFU media without EPO (G/M selective media—GM medium), the frequencies of CFU-G and CFU-GM were affected in 6-day-old EBs only. In 10- and 14-day-old EBs, we did not observe any significant difference in myeloid CFU formation between wt and mutant cells. This is in contrast to CFU-G expansion in complete CFU media, i.e., the full medium (methylcellulose medium with SCF, IL-3, IL-6, and EPO), where we observed a low frequency of CFU-G in mutant cells compared to wt cells. The full medium and GM medium differed only in the presence of EPO. The absence of EPO (GM medium) thus reverses the effect of p38*α* kinase depletion on impaired granulopoiesis.

If granulopoiesis is compensated to a normal level in p38*α*−/− cells due to EPO depletion, we hypothesize that common myeloid progenitors (CMP) and/or their early ancestors undergo transformation into CFU-G. However, in the presence of EPO, they develop normally into CFU-E/BFU-E. This demonstrates that the balance of the EPO level and p38*α* activity determines whether CMP differentiate into erythroid or myeloid lineages. Importantly, this data also suggests a probable difference in EPO signaling. It seems that EPO enables the induction of CFU-E/BFU-E formation in a p38*α*-independent manner but that the maturation of erythroblast is p38*α* dependent. Further detailed study would be required to explain this phenomenon.

The effect of p38 kinase signaling inhibitors (SB203580 and SB202190 [64]) on haematopoiesis in ES cells was also determined. Except for the expressions of c-Kit and Sca1 RNA transcripts, wt cells adopt the phenotype of p38*α*−/− cells in the presence of p38*α* kinase inhibitors (Supp.  3 and 4).

Finally, the potential role of p38*α* in the specification of other germ layers and their progeny should also be taken into consideration. P38*α*−/− ES cells are pluripotent, as are their wt counterpart, and they are able to form all germ layers [10–13, 65]. However, their potential to form some cell lineages appears to be limited. It was reported that the depletion of p38*α in vivo* leads to defects in placental development and the fetus being anemic [14]. Notably, when cells *in vitro* are induced to differentiate by EB formation, p38*α*−/− cells more frequently adopt neuronal phenotypes and the development of mesoderm lineages is attenuated [15, 66]. In agreement with our study, dysregulated mesoderm development might at least partially correlate with the reduced level of Etv2 (expressed in mesoderm progeny) and the low number of hematopoietic CFU observed in 6-day-old EBs. Later, when haematopoiesis is already established and the overall number of hematopoietic CFU is identical in wt and mutant EBs, haematopoiesis might still be affected by p38*α*-dependent phosphorylation and the expression of relevant hematopoietic genes.

In conclusion, in this work, we describe the involvement of p38*α* in a model of the continuous development of haematopoiesis from pluripotent embryonic stem cells *in vitro*. On the basis of our observations, we hypothesized that the depletion of p38*α* regulates the balance in the hematopoietic developmental program by means of several mechanisms. Firstly, we demonstrated that p38*α* is required for the establishment of hemangioblast as a part of Flk1 signaling. Secondly, we showed that p38*α* regulates both the direction of CMP differentiation into the granulocyte lineage and erythroblast maturation. We found that the action of p38*α* is associated with the regulation of the expressions of key transcription factors of haematopoiesis, such as GATA1, Klf1, Runx1, and PU.1, which has also been described previously within particular hematopoietic cell lines or lineages. The most interesting result, the role of p38*α* in the fate of CMP, will require further detailed analysis. Two frequently used inhibitors of p38 kinase signaling also closely mimicked the effect of p38*α* kinase depletion on haematopoiesis, which confirmed that active kinase is required for the regular process. We suggest that, taken together, these findings could help us to understand the role of p38*α* and its importance as a therapeutic target within some leukemic illnesses, as discussed recently [67–70].

## Figures and Tables

**Table 1:** Primers for the particular markers of haematopoiesis used in the study.

Marker	Sequence	Length	Annealing temperature	Used as	Reference
GAPDH	AAGGGCTCATGACCACAGTC	252	62	Reference gene	
	CATACTTGGCAGGTTTCTCCA				
CD34	TGGGTAGCTCTCTGCCTGAT	193	58	Hematopoietic progenitors	[17]
	TGGCTGGTGTGGTCTTACTG				
CD38	CTGTGGTGTGGTCCAAGTGA	248	54,6	Hematopoietic progenitors	[18]
	TGGCAGGCCTGTAGTTATCC				
CD150	GAGAACGTTTCTGTTCAGCAAT	138	61	Marker of hematopoietic progenitors/HSC	[19]
	CTTCACTGTGCAGGCCAACAGC				
C/EBP*α*	CGACTTCTACGAGGTGGAGC	89	61,7	Marker of myelopoiesis	[20]
	GAAAGCCAAAGGCGGCGTTG				
c-Kit	AGTGTGTGGCAGAGGGATTC	274	55,7	Marker HSC	[14]
	GCCTGGATTTGCTCTTTGTTGTTA				
Epo	CCTCATCTGCGACAGTCGAG	79	62	Erythropoietin	[21]
	ACAACCCATCGTGACATTTTCT				
EpoR	GCCCCCTCTGTCTCCTACTT	364	62	Receptor for erythropoietin	[14]
	CGGTGATAGCGAGGAGAACC				
Eklf/Klf1	GGACTTCCTCAAGTGGTGGC	135	61,8	Marker of the switch from embryonic to adult haematopoiesis	[22]
	GTCACGTCCCTCTCATCGTC				
Etv2	AACTAACCACCGAGGTCCCA	170	62,5	TF connected with differentiation of hemangioblast into hematopoietic lineage	[23]
	TCATTCCCGGCTTCCTCTTG				
Flk1 (KDR)	CTACAGACCCGGCCAAACAA	152	54,5	Marker of hemangioblast	[24]
	CAGCTTGGATGACCAGCGTA				
GATA1	GAAGCGAATGATTGTCAGCA	427	61,5	Marker of erythroid cells	[25, 26]
	CAGCAGAGGTCCAGGAAAAG				
GATA2	GGGAGTGTGTCAACTGTGGT	276	61	Marker of hematopoietic progenitors	[27]
	GCCTGTTAACATTGTGCAGC				
G-CSF-R (v1)	TGGCCCTGATGTAGTCTCTCA	149	59,9	Receptor for granulocyte colony-stimulating factor; marker of granulocyte development	[28]
	AAGGCAGGTGGAACACCAGA				
G-CSF-R (v2)	CCGACTGTCAGTACCAAGGG	196	59,9	Receptor for granulocyte colony-stimulating factor; marker of granulocyte development	[28]
	AGTCTGATGGTGGGGGCAAC				
Hbb *γ*	TTGTCCTCTGCTTCTGCCAT	188	57	Embryonic hemoglobin	[26, 29]
	AGCACATTACCCAAGAGTTTG				
Hbb *ζ*	AGAAGGCAGCTATCACAAGCATC	301	59,5	Embryonic hemoglobin	[26, 29]
	CCCAGGAGCTTGAAGTTCTCAGG				
Hbb-b1	GCAGGCTGCTGGTTCTCTACC	165	59,9	Adult hemoglobin	[26, 30]
	TGCCCTTGAGGCTGTCCAAGTGA				
HoxB4	TCACGTGAGCACGGTAAACC	119	62	Marker of haematopoiesis (adult hematopoietic stem cells)	[31, 32]
	CTCCCACCTCTAGCGGGTGC				
IL3	CAGGGAGCAGAACCACGATAA	136	61	Hematopoietic cytokine	[33]
	CCTACAGACCGGATGGAGGA				
IL6	TCTATACCACTTCACAAGTCGGA	88	59	Hematopoietic cytokine	[33]
	GAATTGCCATTGCACAACTCTTT				
M-CSF-R	GGTTGTAGAGCCGGGTGAAA	233	60,9	Receptor for monocyte/macrophage colony-stimulating factor; marker of monocyte/macrophage development	[34, 35]
	AAGAGTGGGCCGGATCTTTG				
PU.1/Spi-1	GTTTCCTACATGCCCCGGAT	178	61,6	Downstream of Runx1	[36]
	TTTTCTTGCTGCCTGTCTCCC				
Runx1/AML1	AGGCAGGACGAATCACACTG	177	57,9	Marker of endothelial-hematopoietic transition	[37, 38]
	CTCGTGCTGGCATCTCTCAT				
Sca1	GGACACTTCTCACACTACAAAG	161	56	Marker of HSC	[17]
	TAACACAGACTCCATCAGGGTAG				
SCL/Tal1	TATGCCCCAGGATGACGGAG	73	60,1	Hematopoietic marker; upstream of Runx1	[24, 39]
	GCGTCCTGTCCCTCTAGTTG				
p38*α*	GATTCTGGATTTTGGGCTGGCTCG	369	55	Verification of lineage	
	ATCTTCTCCAGTAGGTCGACAGCC				

## Data Availability

The data used to support the findings of this study are available from the corresponding author upon request.

## Supplementary Materials

Supplementary Materials Supp. 1. Suggested scheme of haematopoiesis both in *in vivo* (a) [1–3] and in *in vitro* (b) [4, 5] models of ES cell differentiation in mice. The scheme of the potential role of p38 kinase in haematopoiesis based on previous published results and those observed in our work (c). VEGF induces expression of Etv2 in a p38 kinase-dependent manner, which leads to hemangioblast formation and development. EPO/EpoR-induced activation of p38 kinase and phosphorylation (green arrow) of its targets GATA1 and GATA2, in which transcriptional activity is required for erythropoiesis. Haematopoiesis in our experiments with ES cells. The capability of forming hematopoietic colony-forming unit (CFU) progenitors (d), CFU-G, CFU-GM, CFU-M, CFU-E, and CFU-GEMM in differentiating wt ES cells (e). The expression of haematopoiesis-specific transcripts in our model of in vitro haematopoiesis in wt ES cells (f). For details, see Materials and Methods and Results in our manuscript. Supp. 2. Mutated p38*α*−/− cells did not express p38*α* protein, in contrast to their wt counterparts. The expressions of key pluripotent protein Oct4 and generally abundant GAPDH were equal in both cell lines (a). When cells were differentiated by means of EB techniques, the overall level of p38*α* kinase RNA did not change (b). The levels of p38*α* kinase protein as well as its phosphorylated form were also unchanged. This was in contrast to the level of Oct4 protein, a marker of undifferentiated pluripotent cells, that decreased continuously with differentiation time. The protein level of GAPDH, which was used as a reference gene, is also shown (c). The phosphorylated form of p38*α* kinase when wt cells were treated by inhibitors of the p38 kinase pathway SB203580 or SB202190 (5 *μ*M) for 1 hour and subsequently by 200 *μ*M H_2_O_2_ for 1 hour (d). Data are presented as representative Western blot from two experiments and RNA level as *mean* + *SEM* from four independent experiments. Supp. 3. The effect of the p38 kinase pathway inhibitor (SB203580 or SB202190) on the formation of CFU and selected RNA transcripts through EB-differentiating ES cells. The number of CFU and the level of selected RNA transcripts in mutant p38*α*−/− cells are also shown. For details, see Materials and Methods. The concentration of inhibitors was 5 *μ*M. Cells were exposed to inhibitors for the full duration of the differentiation of EBs. Inhibitors were always added to the medium when old medium was replaced by new medium, which was every two days of the culture. The number of all hematopoietic CFU and particular CFU in 6-, 10-, and 14-day-old EBs (a). The expression of RNA transcripts regulating and marking haematopoiesis (b, c) and the expression of RNA transcripts associated with erythropoiesis (d). Data are presented as *mean* + *SEM* from a minimum of three independent experiments. Supp. 4. The table summarizes data of both the effect of p38*α*−/− depletion and the effect of p38 kinase signaling inhibitors on the expression of selected RNA transcripts that are associated with haematopoiesis and that are presented in Figure 3 (for details, see Materials and Methods). It shows that the upregulation (↑), downregulation (↓), and no-change (-) of the mentioned RNA transcript expressions compare to the levels of the same RNA transcripts in wt cells. The c-Kit and Sca1 (both marked in bold in the table) RNA transcripts levels only have an opposite trend in expression in p38*α*−/− cells compared to wt cells treated by p38 kinase inhibitors.
