# Supplementary material for: MAPK p38alpha Kinase Influences Haematopoiesis in Embryonic Stem Cells
Source: Stem Cells Int. 2019 Jun 2;2019:5128135. doi: 10.1155/2019/5128135 (PMC6589316; doi:10.1155/2019/5128135)
Supplement: Supplementary Materials — Supp. 1. Suggested scheme of haematopoiesis both in in vivo (a) [1–3] and in in vitro (b) [4, 5] models of ES cell differentiation in mice. The scheme of the potential role of p38 kinase in haematopoiesis based on previous published results and those observed in our work (c). VEGF induces expression of Etv2 in a p38 kinase-dependent manner, which leads to hemangioblast formation and development. EPO/EpoR-induced activation of p38 kinase and phosphorylation (green arrow) of its targets GATA1 and GATA2, in which transcriptional activity is required for erythropoiesis. Haematopoiesis in our experiments with ES cells. The capability of forming hematopoietic colony-forming unit (CFU) progenitors (d), CFU-G, CFU-GM, CFU-M, CFU-E, and CFU-GEMM in differentiating wt ES cells (e). The expression of haematopoiesis-specific transcripts in our model of in vitro haematopoiesis in wt ES cells (f). For details, see Materials and Methods and Results in our manuscript. Supp. 2. Mutated p38α−/− cells did not express p38α protein, in contrast to their wt counterparts. The expressions of key pluripotent protein Oct4 and generally abundant GAPDH were equal in both cell lines (a). When cells were differentiated by means of EB techniques, the overall level of p38α kinase RNA did not change (b). The levels of p38α kinase protein as well as its phosphorylated form were also unchanged. This was in contrast to the level of Oct4 protein, a marker of undifferentiated pluripotent cells, that decreased continuously with differentiation time. The protein level of GAPDH, which was used as a reference gene, is also shown (c). The phosphorylated form of p38α kinase when wt cells were treated by inhibitors of the p38 kinase pathway SB203580 or SB202190 (5 μM) for 1 hour and subsequently by 200 μM H2O2 for 1 hour (d). Data are presented as representative Western blot from two experiments and RNA level as mean + SEM from four independent experiments. Supp. 3. The effect of the p38 kinase pathway inhibit [file 5128135.f1.pdf]

Supplements

Supp. 1

Suggested scheme of hematopoiesis both *in vivo* (a) [1, 2 ,3] and in *in vitro* model of ES cells differentiation (b) [4, 5] in mice. The scheme of potential role of p38 kinase in hematopoiesis based on previous published results and observed in our work (c). VEGF induces expression of Etv2 in p38 kinase-dependent manner, what leads to hemangioblast formation and development. EPO/EpoR induced activation of p38 kinase and phosphorylation (green arrow) of its targets GATA1 and 2, which transcriptional activity is required for erythropoiesis. Hematopoiesis in our experiments with ES cells. The capability forming hematopoietic colony forming unit (CFU) progenitors (d), CFU-G, -GM, -M, -E, and -GEMM in differentiating wt ES cells (e). The expression of hematopoiesis specific transcripts in our model of in vitro hematopoiesis in wt ES cells (f). For details, see Materials and Methods, and Results in our manuscript.

Supp. 2

Mutated p38α-/- cells did not express p38α protein, in contrast to their wt counterparts. The expression of key pluripotent protein Oct4 and general abundant GAPDH was equal in both cell lines (a). When cells were differentiated by means of EB techniques, the overall level of p38α kinase RNA did not change (b). The level of p38α kinase protein as well as its phosphorylated form were also unchanged. This was in contrast to the level of Oct4 protein, a marker of undifferentiated pluripotent cells, that decreased continuously with differentiation time. The protein level of GAPDH, which was used as a reference gene, is also shown (c). The phosphorylated form of p38α kinase when wt cells were treated by inhibitors of p38 kinase pathway SB203580 or SB202190 (5μM) for 1 hour and subsequently by 200 μM H_2O_2 for 1 hour (d). Data are presented as representative western blot from two experiments, RNA level as mean + SEM from four independent experiments.

Supp. 3

The effect of p38 kinase pathway inhibitor (SB203580 or SB202190) on the formation of CFU and selected RNA transcripts through EB-differentiating ES cells. The number of CFU and the level of selected RNA transcripts in mutant p38α-/- cells are also shown. For details, see Materials and Methods. The concentration of inhibitors was 5 μM. Cells were exposed to inhibitors for the full duration of the differentiation of EBs. Inhibitors were always added to the medium when old medium was replaced by new medium, which was every two days of the culture. The number of all hematopoietical CFU and particular CFU in 6-, 10-, and 14-day-old EBs (a). The expression of RNA transcripts regulating and marking hematopoiesis (b, c) and the expression of RNA transcripts associated with erythropoiesis (d). Data are presented as mean + SEM from a minimum of three independent experiments.

Supp. 4

The table summarizes data both the effect of p38α-/- depletion and the effect of p38 kinase signaling inhibitors on the expression of selected RNA transcripts that are associated with hematopoiesis and that are presented on Fig. 3 (For details, see Material and Methods). It shows that the up-regulation (↑), down-regulation (↓) and no-change (-) of the mentioned RNA transcript expressions compare to the levels of the same RNA transcripts in wt cells. The cKit and Sca1 (both marked in bold in the table) RNA transcripts levels only have an opposite trend in expression in p38α-/- cells compared to wt cells treated by p38 kinase inhibitors.

	6ds old Ebs			10ds old Ebs			14ds old Ebs		
Marker	p38α-/-	SB203580	SB202190	p38α-/-	SB203580	SB202190	p38α-/-	SB203580	SB202190
CD34	↓	-	-	↓	↓	↓	↓	↓	↓
CD38	-	↓	-	↓	↓	↓	↓	↓	↓
**cKit**	↑	↓	↓	↑	↓	↓	↑	↓	↓
Flk1	↓	↓	↓	↓	↓	↓	↓	-	↓
Hbb-b1	↓	↓	↓	↓	↓	↓	↓	↓	↓
Hbbζ	↓	↓	↓	↓	↓	↓	↓	↓	↓
Hbbγ	↓	↓	↓	↓	↓	↓	↓	↓	↓

HoxB4	-	-	-	-	-	-	-	-	-
GATA1	↓	↓	↓	↓	↓	↓	↓	↓	↓
GATA2	-	-	-	-	-	-	↓	↓	↓
Klf1	-	-	-	↓	↓	↓	↓	↓	↓
PU.1	-	-	-	-	↓	-	↓	↓	↓
Runx1	-	-	-	-	-	-	↓	↓	↓
**Sca1**	↑	↓	↓	↑	↓	↓	-	-	-
VEGF	-	-	-	-	-	-	-	↓	↓

References

1. T.L. Huber, V. Kouskoff, H.J. Fehling, J. Palis, and G. Keller, “Haemangioblast commitment is initiated in the primitive streak of the mouse embryo.” *Nature*. vol. 432, no. 7017, pp. 625–630, 2004.

2. A. Medvinsky, S. Rybtsov, and S. Taoudi, “Embryonic origin of the adult hematopoietic system: advances and questions.” *Development*. vol. 138, no. 6, pp. 1017–1031, 2011.

3. T.C. Doetschman, H. Eistetter, M. Katz, W. Schmidt, and R. Kemler, “The in vitro development of blastocyst-derived embryonic stem cell lines: formation of visceral yolk sac, blood islands and myocardium.” *J. Embryol. Exp. Morphol.* vol. 87, pp. 27–45, 1985.

4. G. Keller, M. Kennedy, T. Papayannopoulou, and M. V Wiles, “Hematopoietic commitment during embryonic stem cell differentiation in culture.” *Mol. Cell. Biol.* vol. 13, no. 1, pp. 473–486, 1993.

5. M. V Wiles and G. Keller, “Multiple hematopoietic lineages develop from embryonic stem (ES) cells in culture.” *Development*. vol. 111, no. 2, pp. 259–267, 1991.

Supplement 1.

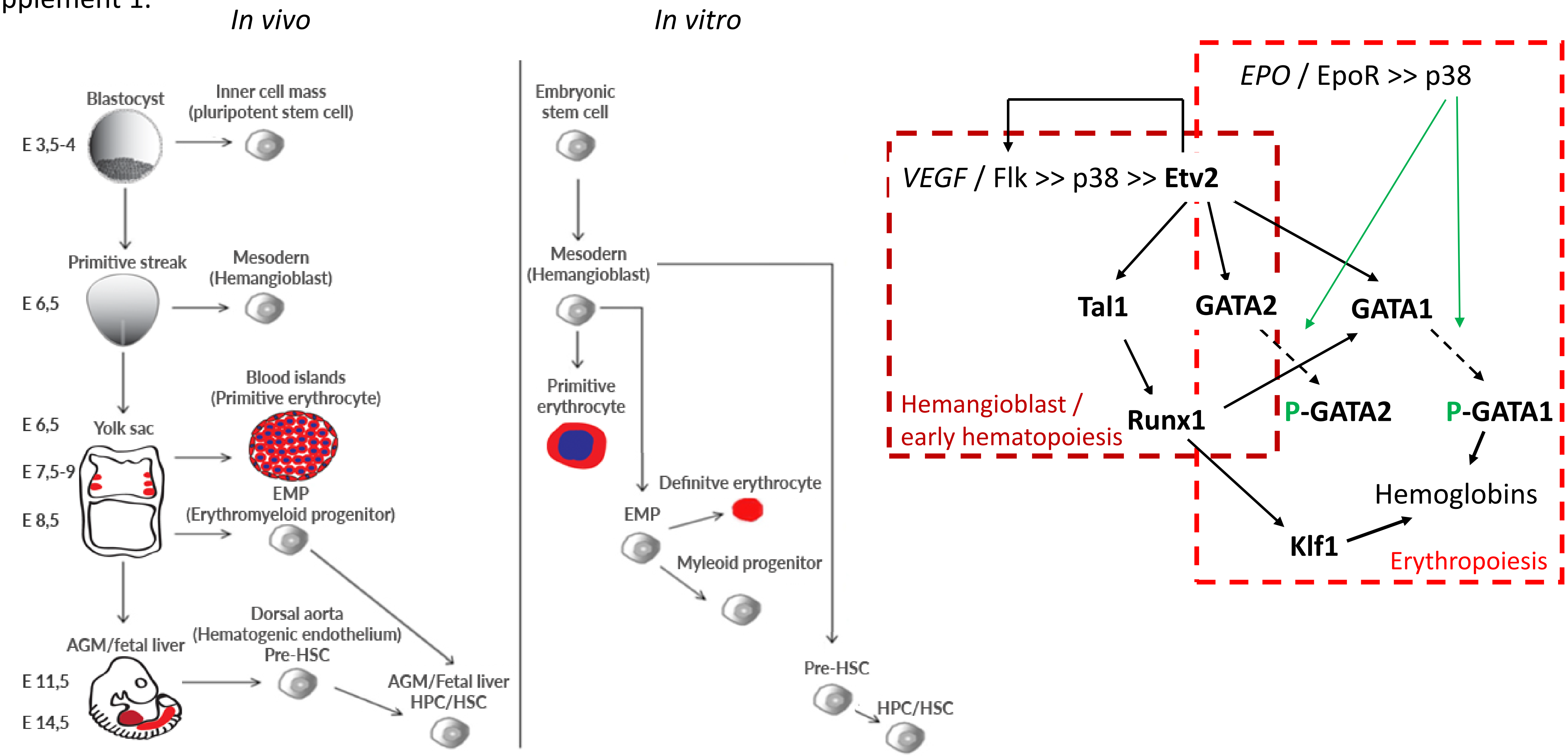

(a) (b) (c)

Supplement 1.

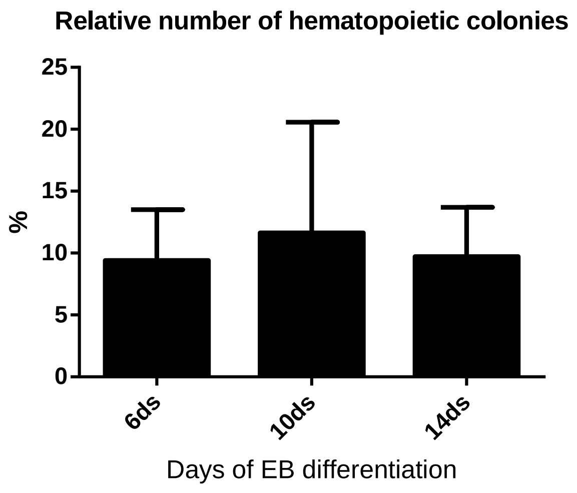

(d)

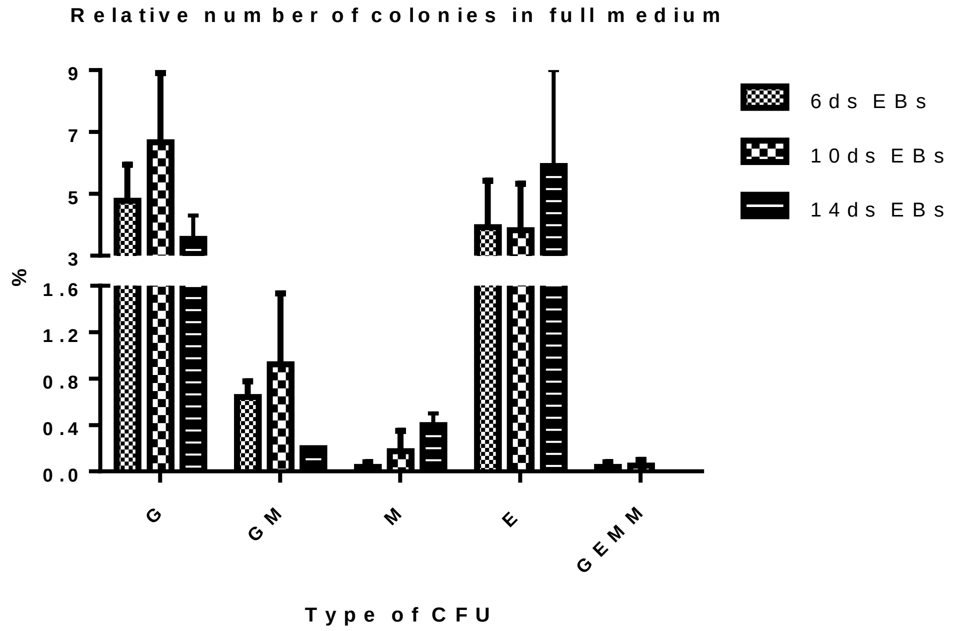

(e]

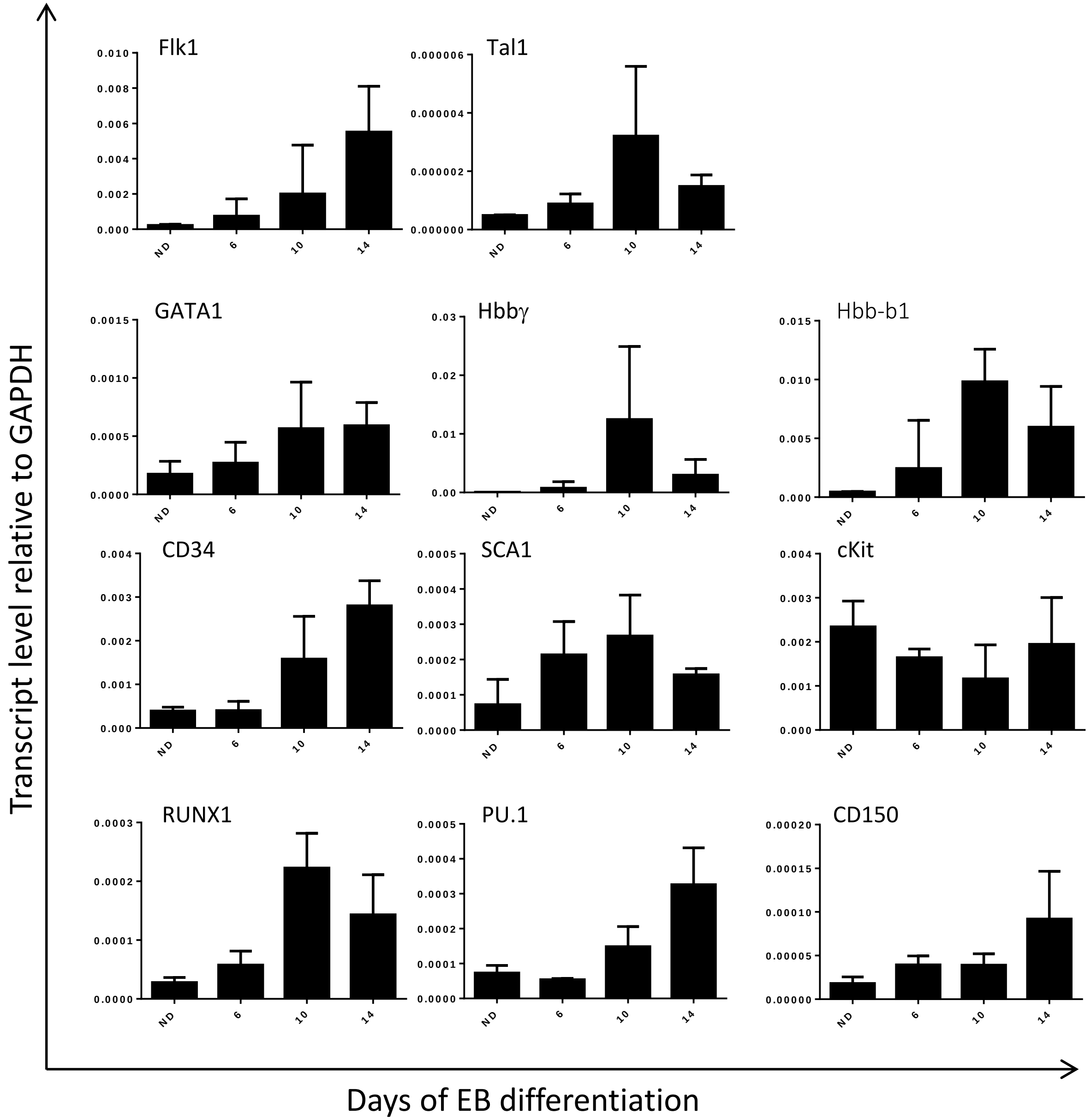

(f)

Supplement 2.

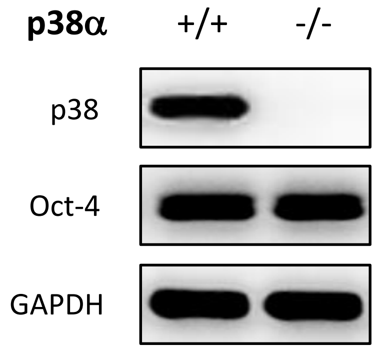

(a)

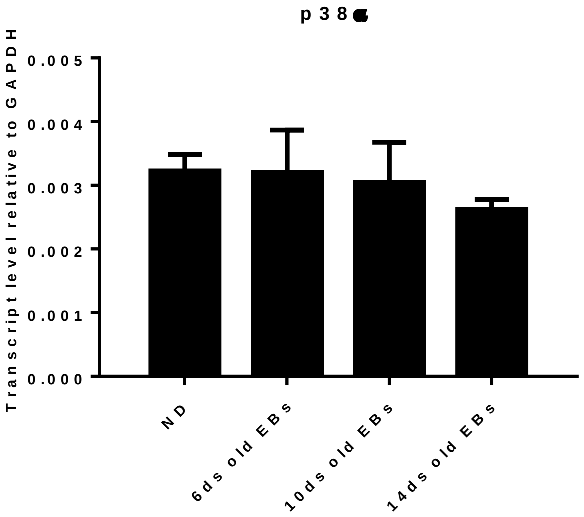

(b)

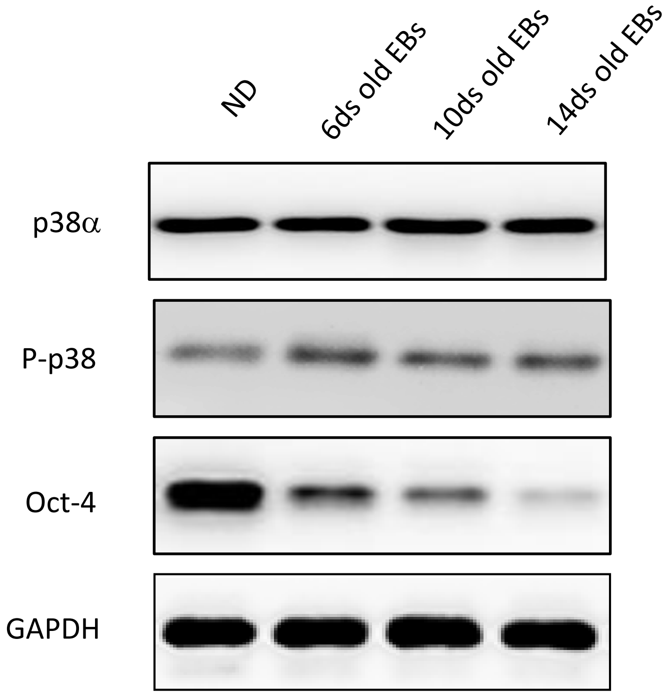

(c)

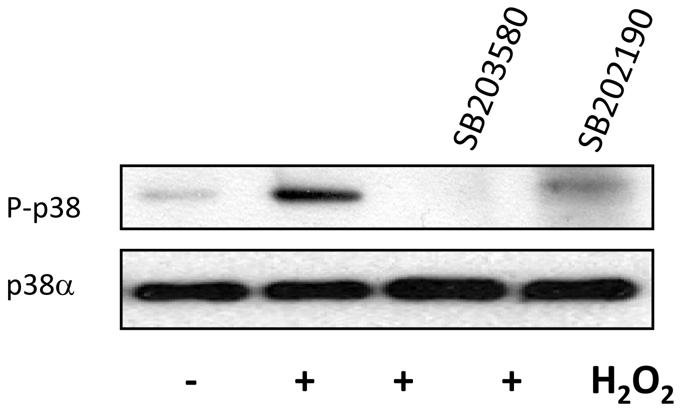

(d)

Supplement 3.

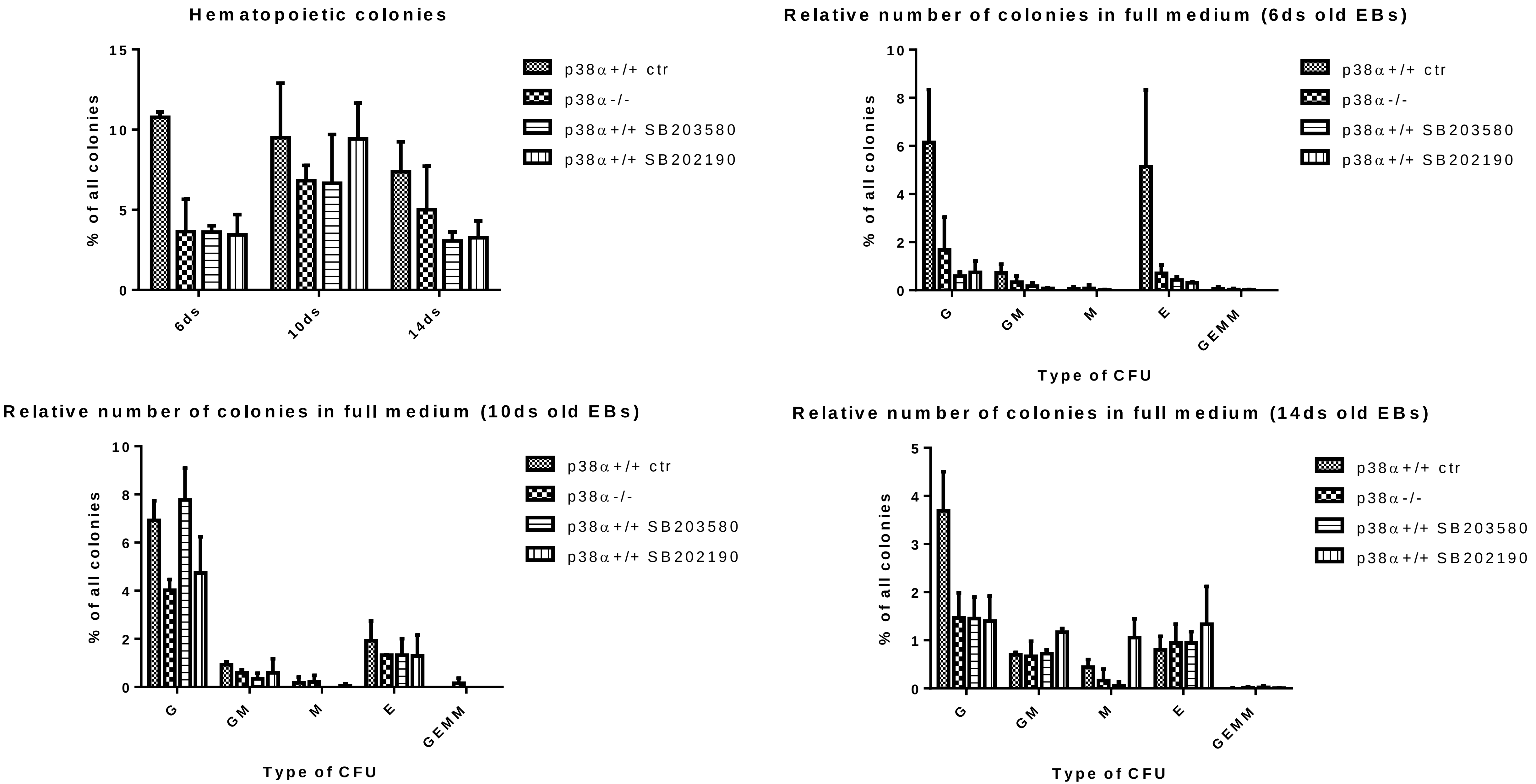

(a)

Supplement 3.

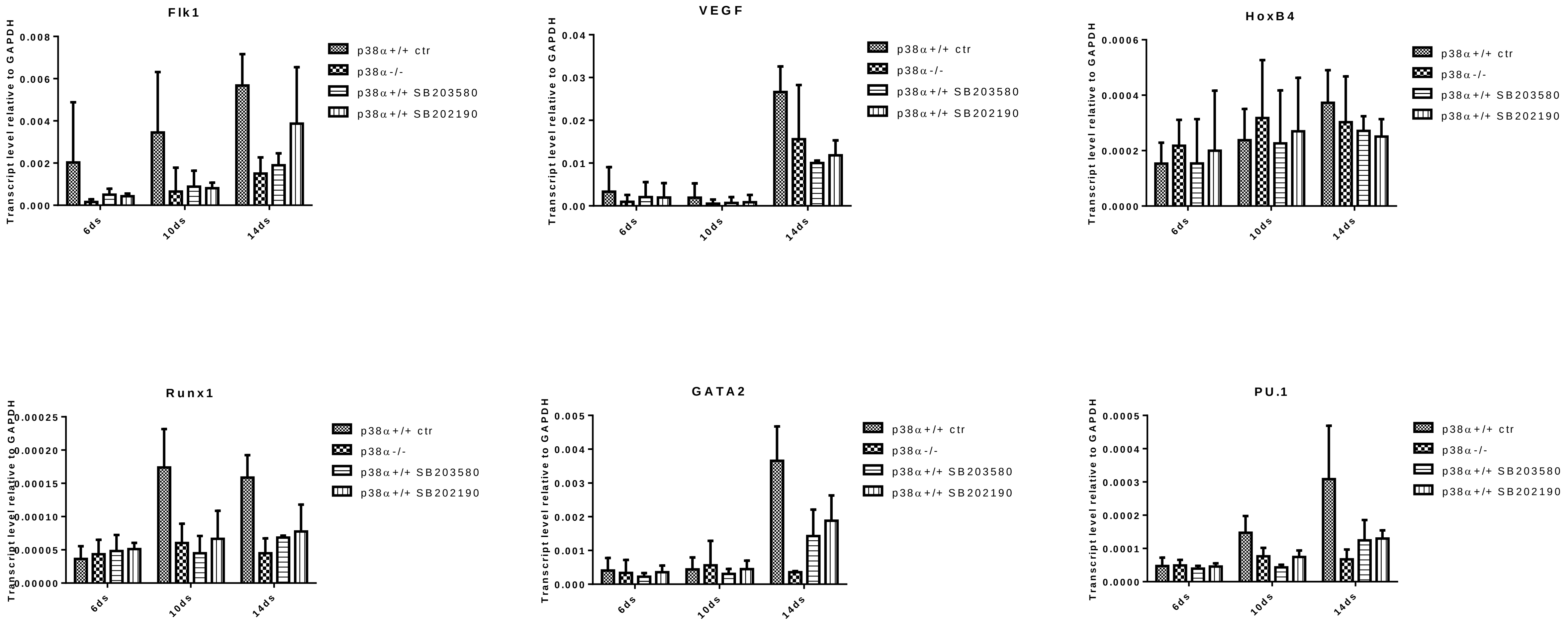

(b)

Supplement 3.

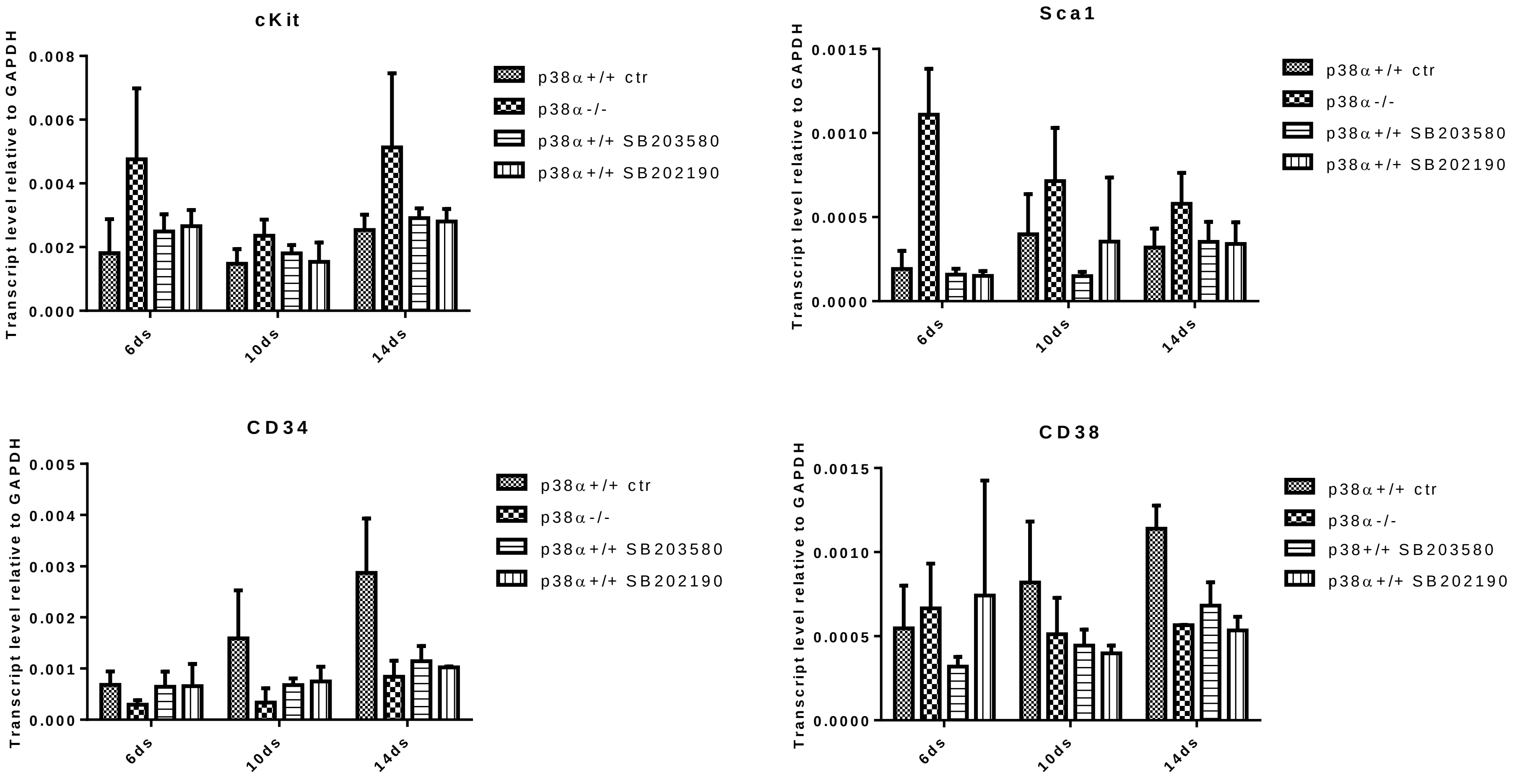

(c)

Supplement 3.

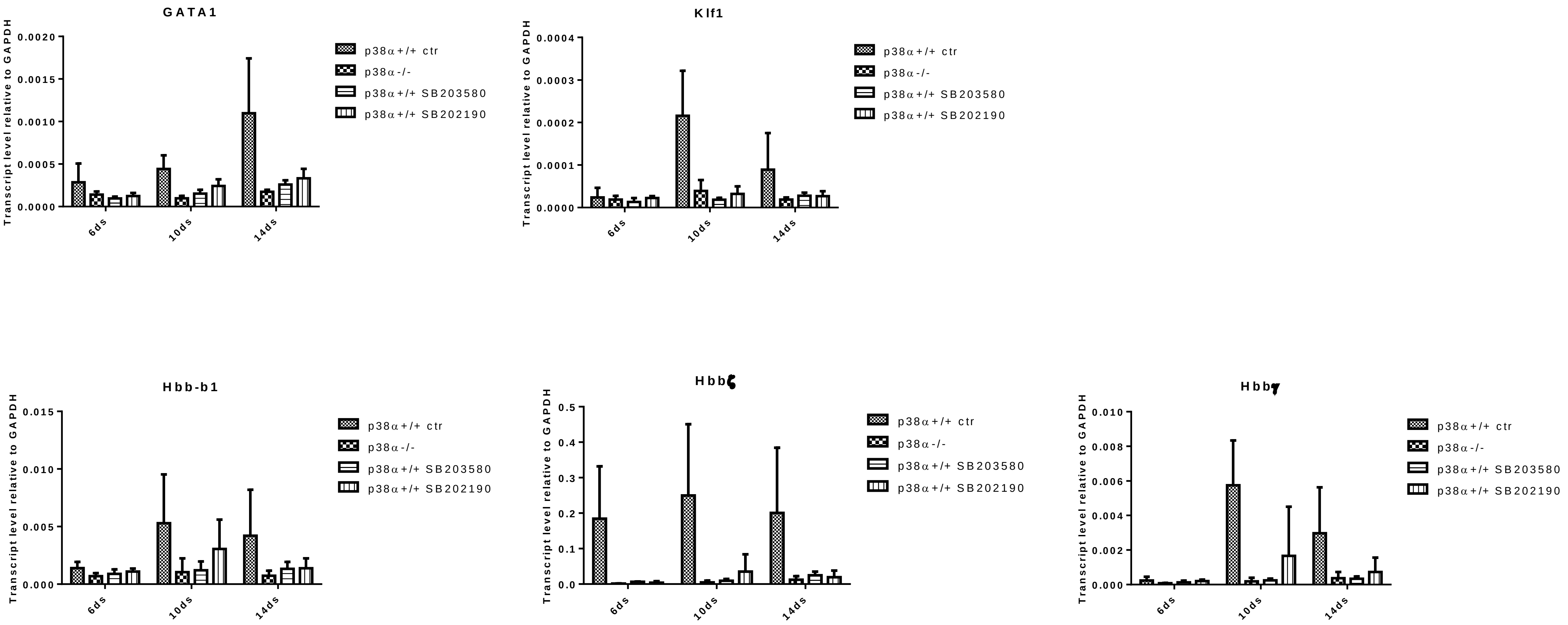

(d)
